# Bone mesenchymal stem cells stimulation by magnetic nanoparticles and a static magnetic field: release of exosomal miR-1260a improves osteogenesis and angiogenesis

**DOI:** 10.1186/s12951-021-00958-6

**Published:** 2021-07-13

**Authors:** Di Wu, Xiao Chang, Jingjing Tian, Lin Kang, Yuanhao Wu, Jieying Liu, Xiangdong Wu, Yue Huang, Bo Gao, Hai Wang, Guixing Qiu, Zhihong Wu

**Affiliations:** 1 Department of Orthopaedic Surgery, Peking Union Medical College Hospital, Peking Union Medical College and Chinese Academy of Medical Sciences, No.1 Shuaifuyuan, Beijing, 100730 China; 2 Medical Science Research Center (MRC), Peking Union Medical College Hospital, Peking Union Medical College and Chinese Academy of Medical Sciences, No.1 Shuaifuyuan, Beijing, 100730 China; 3 Umibio (Shanghai) Co. Ltd; RM309, 1st building, No.88 Cailun Rd, Shanghai, 201210 China; 4 Beijing Key Laboratory for Genetic Research of Bone and Joint Disease, No.1 Shuaifuyuan, Beijing, 100730 China; 5 State Key Laboratory of Complex Severe and Rare Diseases, Peking Union Medical College Hospital, No.1 Shuaifuyuan, Beijing, 100730 China

**Keywords:** Magnetic nanoparticles, Static magnetic field, miR-1260a, Exosomes, Bone regeneration

## Abstract

**Background:**

The therapeutic potential of exosomes derived from stem cells has attracted increasing interest recently, because they can exert similar paracrine functions of stem cells and overcome the limitations of stem cells transplantation. Exosomes derived from bone mesenchymal stem cells (BMSC-Exos) have been confirmed to promote osteogenesis and angiogenesis. The magnetic nanoparticles (eg. Fe_3_O_4_, γ-Fe_2_O_3_) combined with a static magnetic field (SMF) has been commonly used to increase wound healing and bone regeneration. Hence, this study aims to evaluate whether exosomes derived from BMSCs preconditioned with a low dose of Fe_3_O_4_ nanoparticles with or without the SMF, exert superior pro-osteogenic and pro-angiogenic activities in bone regeneration and the underlying mechanisms involved.

**Methods:**

Two novel types of exosomes derived from preconditioned BMSCs that fabricated by regulating the contents with the stimulation of magnetic nanoparticles and/or a SMF. Then, the new exosomes were isolated by ultracentrifugation and characterized. Afterwards, we conducted in vitro experiments in which we measured osteogenic differentiation, cell proliferation, cell migration, and tube formation, then established an in vivo critical-sized calvarial defect rat model. The miRNA expression profiles were compared among the exosomes to detect the potential mechanism of improving osteogenesis and angiogenesis. At last, the function of exosomal miRNA during bone regeneration was confirmed by utilizing a series of gain- and loss-of-function experiments in vitro.

**Results:**

50 µg/mL Fe_3_O_4_ nanoparticles and a 100 mT SMF were chosen as the optimum magnetic conditions to fabricate two new exosomes, named BMSC-Fe_3_O_4_-Exos and BMSC-Fe_3_O_4_-SMF-Exos. They were both confirmed to enhance osteogenesis and angiogenesis in vitro and in vivo compared with BMSC-Exos, and BMSC-Fe_3_O_4_-SMF-Exos had the most marked effect. The promotion effect was found to be related to the highly riched miR-1260a in BMSC-Fe_3_O_4_-SMF-Exos. Furthermore, miR-1260a was verified to enhance osteogenesis and angiogenesis through inhibition of HDAC7 and COL4A2, respectively.

**Conclusion:**

These results suggest that low doses of Fe_3_O_4_ nanoparticles combined with a SMF trigger exosomes to exert enhanced osteogenesis and angiogenesis and that targeting of HDAC7 and COL4A2 by exosomal miR-1260a plays a crucial role in this process. This work could provide a new protocol to promote bone regeneration for tissue engineering in the future.

**Graphical abstract:**

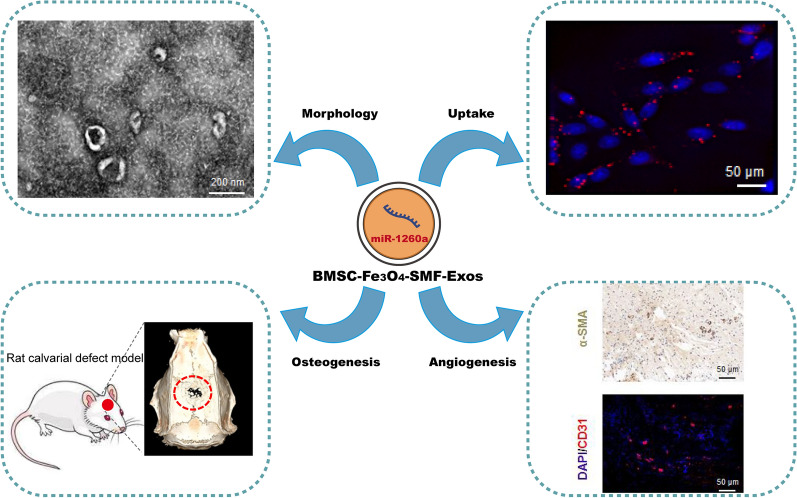

**Supplementary Information:**

The online version contains supplementary material available at 10.1186/s12951-021-00958-6.

## Background

Treatment of large bone defects, secondary to trauma, tumor, or infection, is still a great challenge for orthopaedic surgeons. Because it may cause severely delayed union or even nonunion and compromise the musculoskeletal function of patients [1, 2]. Currently, the standard clinical treatments for bone restoration are autologous and allogeneic transplantation [3]. But they have several limitations to restrict their widespread applications, such as limited supply, complications at donor sites, risks of disease transmissions, and so on [4, 5]. Bone tissue engineering, including scaffolds, cells and growth factors, offers a promising approach to overcome these limitations and draws more attentions from researchers in recent years [6, 7].

Angiogenesis, blood vessel growth, has been recognized to play an essential role in remodeling new bone (osteogenesis) [8]. Oxygen and nutrition supply are limited to no more than 200 µm through diffusion [9]. Cells will not survive and new bone formation will be hindered in the center of the bone defect without vessel networks. Thus, it is imperative to improve osteogenic and angiogenic activities simultaneously during bone repair, using the techniques of tissue engineering with scaffolds, various cells, and/or biological factors [10].

Regarding the cellular component of tissue engineering, bone mesenchymal stem cells (BMSCs) are attractive potential therapeutic agents for the promotion of osteogenesis and angiogenesis in bone defect repair [11]. BMSCs are easily obtained from donors; transplantation of these cells results in a low incidence of graft-versus-host disease, and they possess osteogenic properties. However, direct transplantation of stem cells still has some challenges and limitations in the clinic, such as consuming time, large cells demand, a low survival rate of transplanted cells, the risk of tumor formation and immune rejection [12].

Exosomes derived from stem cells are generally small (30 to 150 nm) membrane particles of endosomal origin whose contents are protected from degradation; they deliver a variety of small biomolecules, including mRNAs, miRNAs and proteins, to recipient cells [13, 14]. Recent evidence indicates that stem cells play important roles in tissue regeneration mainly through a paracrine mechanism, and exosomes are important contributors to these paracrine functions [15]. Exosomes exhibit stem cell-like proregenerative properties, and direct treatment with exosomes may avoid many of the adverse effects of stem cell transplantation therapy. Most importantly, exosomes do not contain MHC-I or MHC-II proteins and thus overcome the disadvantages of cell transplantation therapy and seldom induce overt immune reactions [16]. Previous studies have demonstrated that exosomes derived from BMSCs (BMSC-Exos) can exhibit similar therapeutic functions with those of BMSCs in the treatment for bone regeneration by delivering exosomes [17, 18].

Magnetic nanoparticles (MNPs), such as γ-Fe_2_O_3_ and Fe_3_O_4_, have great potential applications in bone tissue engineering, and can promote osteogenesis and angiogenesis with or without a static magnetic field (SMF) [19–21]. Mechanotransduction, the conversion of continuous weak magnetic forces acting on a cell to internal biochemical signals, is its most likely mechanism to enhance bone regeneration [22–25]. Bambini et al. found more newly formed bone volumes around dental implants inserted in the tibia of rabbits after SMF stimulation, thus the SMF enhanced bone healing [26]. Another study also revealed that SMFs improved bone regeneration around dental implants through micro-CT, histology, microarrays, and real-time PCR [27]. The physical mechanisms of SMF and its biologic effects may include electrodynamic interactions, magnetomechanical interactions, and effects on electronic spin states. And our previous study found SMFs enhanced osteogenic differentiation through up-regulating Smad4 [28].

Additionally, low doses of MNPs are safe and have been approved by the Food and Drug Administration (FDA) to treat iron-deficiency anemia, because they can be assimilated through ionization into iron ions and participate in iron homeostasis [29, 30]. Lee et al. injected iron oxide nanoparticles (IONPs) into the infarcted heart and magnetic guidance reduced apoptosis and fibrosis, and enhanced angiogenesis and cardiac function recovery [31]. Zhu et al. revealed the regulatory roles of magnetic signals of hydroxyapatite scaffold in osteoblast-osteoclast communication via exosomes [32]. Recently, Kim et al. showed magnetic nanovesicles (MNV) derived from IONP-harboring BMSC can improve the ischemic-lesion targeting efficiently, because MNV contained greater amounts of those therapeutic molecules compared to nanovesicles derived from naive BMSC [33].

Stress inducing stimuli and activation of cells can regulate the contents of exosomes [34]. Additionally, lots of studies have reported that exosomes from stem cells can promote osteogenesis and angiogenesis [35]. For example, Qin et al. summarized the recent reports using exosomes to regulate osteogenesis and accelerate bone regeneration [36]. Our recent study showed that exosomes derived from BMSCs cultured under magnetic conditions enhanced wound healing and the underlying mechanism was elucidated [37]. To further enhance the effects of BMSC-Exos for bone regeneration, we synthesized a new type of exosomes derived from BMSCs treated with the stimulations of MNPs (Fe_3_O_4_ nanoparticles) and/or a SMF (BMSC-Fe_3_O_4_-Exos or BMSC-Fe_3_O_4_-SMF-Exos) in this study. Their osteogenic and angiogenic activities were then assessed compared with BMSC-Exos and control groups using BMSCs and human umbilical vein endothelial cells (HUVECs) in vitro and a critical-sized calvarial defect rat model in vivo. Finally, the potential molecular mechanisms for improving bone regeneration were explored by sequencing exosomal miRNAs.

## Materials and methods

### Cell culture

Human BMSCs (ATCC; Manassas, VA, USA) were cultured in basal medium (HUXMA-90011, Cyagen Biosciences, Santa Clara, CA, USA) containing 10% fetal bovine serum (FBS). HUVEC and HEK-293 cells were obtained from the cell bank of the Chinese Academy of Medical Sciences (Beijing, China) and cultured in high-glucose Dulbecco's modified Eagle’s medium (DMEM, Gibco BRL, Grand Island, NY, USA) containing 10% FBS and 1% penicillin–streptomycin.

### Magnetic Fe_3_O_4_ and SMF

**Fig. 1:**
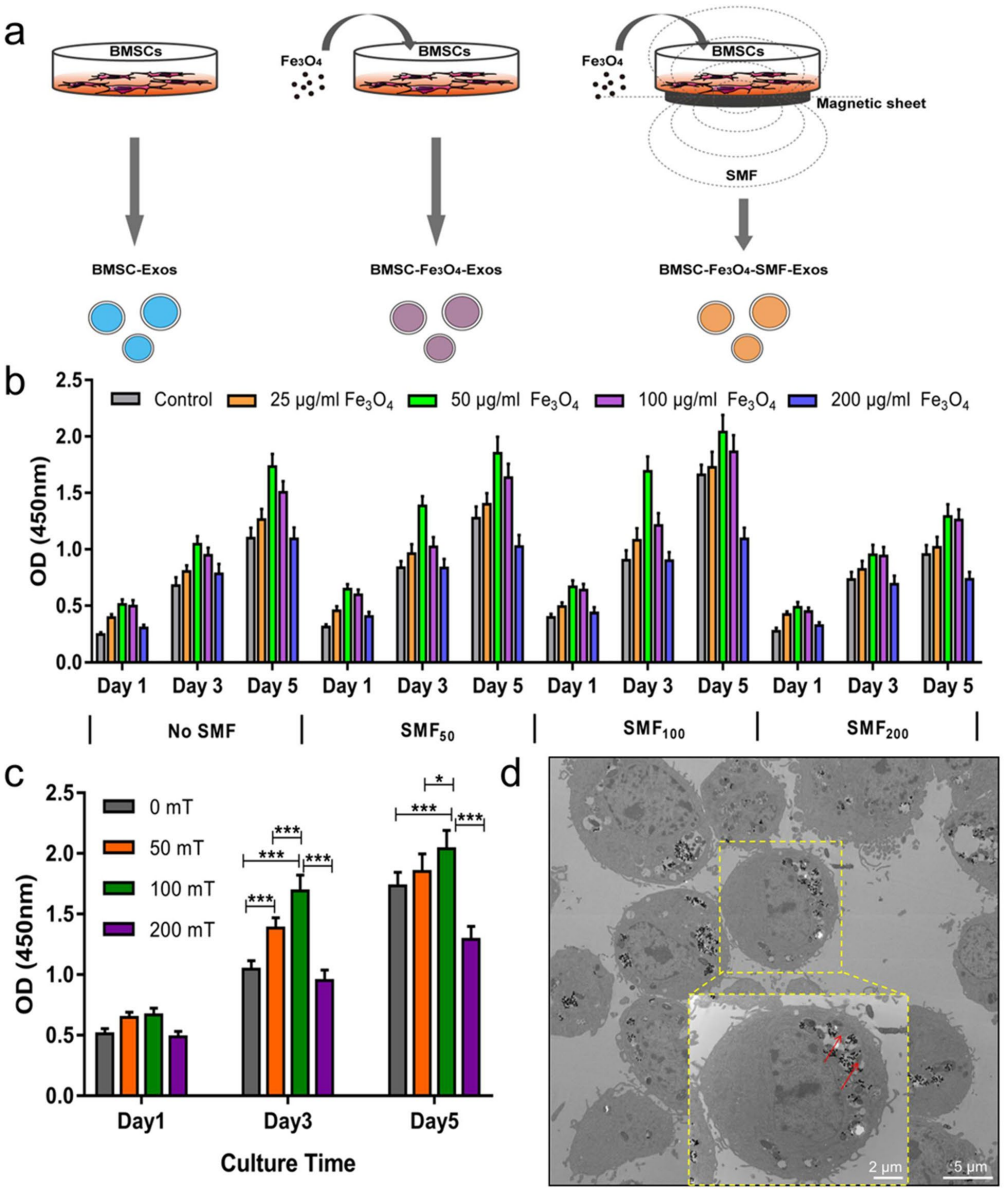
Fabrication of three types of exosomes: BMSC-Exos, BMSC-Fe_3_O_4_-Exos and BMSC-Fe_3_O_4_-SMF-Exos. (*) *p* < 0.05, (***) *p* < 0.001. **a** Schematic illustration of magnetic stimulation using Fe_3_O_4_ nanoparticles and SMF. **b** CCK8 assay for the proliferation of BMSCs cultured in the presence of various concentrations of Fe_3_O_4_ nanoparticles combined with exposure to SMFs of different strengths. **c** Proliferation of BMSCs treated with the optimal working concentration (50 µg/mL) of Fe_3_O_4_ nanoparticles and exposed to SMFs of different strengths. **d** TEM images of the Fe_3_O_4_ and BMSCs. The dotted yellow box indicates the internalization of Fe_3_O_4_ by BMSCs, and the red arrows indicate the Fe_3_O_4_ nanoparticles

10 mg Fe_3_O_4_ nanoparticles powder (Nanjing Nanoeast Biotech, Jiangsu, China) was first soaked in 50 mL basal medium (200 µg/mL) with shaking at 37 °C for 24 h, and the concentrations of Fe_3_O_4_ in the medium were then diluted to 100, 50 and 25 µg/mL in sequence. The size of Fe_3_O_4_ nanoparticles is 100 nm, with high magnetic responsiveness, uniform particle size distribution, and great stability, biocompatibility and solubility. The SMF environments for culturing BMSCs were set to 0, 50, 100, and 200 mT using a magnet (10 cm diameter), which composed of lots of monolayer small magnetic sheets (2 mm thick ×10 mm diameter; Shenzhen Strong Magnets, Guangdong, China), under the culture wells by controlling the distance or increasing/decreasing the small magnetic sheets, respectively. The intensities of SMF were measured by a Gauss meter (TM-701, Kanetec, Tokyo, Japan). BMSCs were separately cultured in medium alone or medium containing Fe_3_O_4_ nanoparticles at various concentrations with or without exposure to a SMF (Fig. 1a). The morphology of BMSCs after engulfing Fe_3_O_4_ nanoparticles was observed by transmission electron microscopy (TEM, Hitachi, Tokyo, Japan).

### Cell proliferation assay

The proliferation of BMSCs was measured using the Cell Counting Kit-8 assay (CCK-8; Dojindo, Tokyo, Japan). BMSCs (5 × 10^3^ cells/well) were seeded in the medium containing various concentrations of Fe_3_O_4_ nanoparticles (200, 100, 50, 25, and 0 µg/mL) combined with exposure to different SMF conditions (0, 50, 100, and 200 mT) using 96-well plates (each group: n = 4). After culturing for 1, 3, or 5 days, the CCK-8 reagent was added to the medium, and the absorbance at 450 nm was measured 2 h later.

### Exosome isolation and purification

BMSCs were washed and incubated for 48 h in the complete medium supplemented with 10% exosome-depleted FBS (Umibio, Shanghai, China) when they reached 70 − 80% confluence. The supernatant was collected using centrifugation (300×*g* for 10 min and 2000×*g* for 20 min) to remove cell debris. Then, the suspension was centrifuged at 10,000 × g for 30 min, followed by ultracentrifugation for 70 min at 100,000×*g* after it was filtered using a 0.22-μm filter (Merck-Millipore, Darmstadt, Germany). At last, the pelleted exosomes were washed twice with a large volume of PBS and centrifuged again at 110,000×*g* for 70 min to remove contaminating protein. All these procedures were performed at 4 °C, and the prepared exosomes were resuspended in PBS and stored at − 80 °C.

### Exosome identification and internalization

The size distribution of exosomes was measured using the NanoSight NS500 instrument (Malvern Instruments, Malvern, UK) through the nanoparticle tracking analysis (NTA) software, and the morphology of exosomes was observed by TEM according to Raposo’s descriptions [38]. The exosomes were verified with specific exosome surface markers by western blotting, including CD9, CD63, CD81, and TSG101.

To measure the endocytosis of exosomes by BMSCs or HUVECs, exosomes were first labeled with the red fluorescent dye PKH26 (Sigma-Aldrich, Darmstadt, Germany) according to the manufacturer's instructions [39]. Then, they were incubated with BMSCs or HUVECs for 4, 8, 12, 16 and 24 h at 37 °C. Finally, the fluorescence intensities of PKH26 were measured using a confocal microscopy (Nikon, Tokyo, Japan).

### Osteogenic differentiation

Osteogenic differentiation of BMSCs was initiated 24 h after incubation of the cells with exosomes or transfection of the cells with miRNA mimics or inhibitors. Briefly, the original medium was replaced with an osteogenic differentiation medium (HUXMA-90021, Cyagen Biosciences) containing exosome-depleted FBS, penicillin–streptomycin, dexamethasone, ascorbic acid, glutamine, and β-glycerophosphate. The differentiation medium was refreshed every 72 h together with the addition of 200 μL of either PBS, BMSC-Exos (100 µg/mL), BMSC-Fe_3_O_4_-Exos (100 µg/mL) or BMSC-Fe_3_O_4_-SMF-Exos (100 µg/mL). Transfection with mimics or inhibitors was performed after the differentiation medium was refreshed. Total RNA was extracted 7 and 14 days after differentiation for qRT-PCR analysis.

To assess the mineralization, Alizarin Red staining (ARS) was performed on day 14 of osteoinduction. The cells were stained with 2% ARS solution (Sigma-Aldrich) for 10 min and then washed with distilled water. To quantitatively determine matrix calcification, the cells were destained in 10% cetylpyridinium chloride in 10 mM sodium phosphate for 30 min and evaluated by measuring their absorbance at 562 nm. Alkaline phosphatase (ALP) activity was measured using an ALP assay kit (Beyotime, Jiangsu, China) on days 7 and 14 of culture in osteogenic differentiation medium. The BMSCs were lysed using 0.1% Triton X-100 and Tris–HCl for 2 h at 4 °C. The p-nitrophenyl phosphate and lysate were mixed and incubated at 37 °C for 15 min. The measured ALP activity was normalized to the total intracellular protein content, which was measured using a Pierce BCA Protein Assay Kit (Thermo Fisher Scientific, Waltham, MA, USA).

### Scratch wound and transwell assays

HUVECs were first cultured in 6-well plates until confluent to perform the wound healing assay. The monolayer was scratched using a 200-μL pipette tip and detached cells were rinsed out with PBS. The residual cells were cultured in different serum-free medium containing 200 μL PBS, 200 μL BMSC-Exos (100 µg/mL), 200 μL BMSC-Fe_3_O_4_-Exos (100 µg/mL) or 200 μL BMSC-Fe_3_O_4_-SMF-Exos (100 µg/mL), respectively. Images of HUVECs were separately captured at the beginning and after 24 h. The migration rate of cells was calculated as follows: migration rate (%) = (A_0_–A_n_)/A_0_ × 100%, where A_0_ represents the area of the initial wound and A_n_ represents the remaining area of the wound at the measurement point.

HUVECs (1 × 10^5^ cells/well) were suspended in serum-free medium and seeded in the upper chambers of 24-well transwell plates with 8-μm pore filters (Corning, NY, USA) to perform the transwell assay. Then, four groups were set as follows: 200 μL negative control (PBS), 200 μL BMSC-Exos (100 µg/mL), 200 μL BMSC-Fe_3_O_4_-Exos (100 µg/mL) or 200 μL BMSC-Fe_3_O_4_-SMF-Exos (100 µg/mL) were added to the wells, respectively. Each lower chamber was filled with DMEM supplemented with 10% exosome-depleted FBS. After incubation at 37 °C for 24 h, the cells on the lower surface were stained with 0.1% crystal violet for several minutes after removing the attached cells on the upper surface of the filter membranes. The extent of cell migration was observed using the optical microscope (Leica, Solms, Germany).

### Tube formation assay

The tube formation assay was conducted to evaluate angiogenesis in vitro using a Matrigel basement membrane matrix (356,234, BD Biosciences, San Jose, CA, USA) according to the manufacturer's instructions. Briefly, the matrigel (50 μL/well) was added to 96-well plates using cold pipette tips on ice after it was thawed overnight at 4 °C. Then, the plates were incubated at 37 °C until the Matrigel became solidified. Next, HUVECs (5 × 10^4^ cells/well) were seeded in the complete medium, supplementing with 10% exosome-depleted FBS. Subsequently, 10 μL PBS, 10 μL BMSC-Exos (100 µg/mL), 10 μL BMSC-Fe_3_O_4_-Exos (100 µg/mL) or 10 μL BMSC-Fe_3_O_4_-SMF-Exos (100 µg/mL) were added, respectively. Tube formations were evaluated using an inverted microscope after they were incubated at 37 °C for 6 h. The total length of the tubes was measured using ImageJ software (each well: n = 5, Media Cybernetics, Bethesda, MD, USA).

### qRT‑PCR analysis

Total RNA of the cells was isolated using Trizol (Invitrogen, Carlsbad, CA, USA), and then reverse-transcribed using a Revert Aid first-strand cDNA synthesis kit (Takara, Shiga, Japan) according to the instructions. MiRNA expression was assessed using a SYBR Green microRNA assay kit (Applied Biosystems, Foster City, CA, USA) after exosomal miRNAs were extracted using the Exosome RNA Purification Kit (Umibio, Shanghai, China). qRT-PCR was performed on the ABI PRISM 7900HT System using the SYBR Green-based real-time detection method. GAPDH and U6 were used to normalize the mRNA and miRNA expression levels, and the 2^−ΔΔCt^ approach was used for relative quantification of the mRNA and miRNA expressions. The PCR primer sequences were listed in Additional file 1: Tables S1, S2.

### Western blotting

The concentrations of total proteins in cells or exosomes were measured using a BCA protein assay kit (Thermo Fisher Scientific). Proteins were transferred to PVDF membranes and probed with the appropriate primary antibodies (1:1000) after they were separated via 10% SDS-PAGE. Secondary antibodies were added to probe the blots, and the immunoreactive bands were visualized with chemiluminescence reagents (Thermo Fisher Scientific) and quantified using ImageJ software. The primary antibodies were obtained from Cell Signaling Technology, Beverly, MA, USA (CD9, CD63, CD81, TSG101, and Calnexin), or Abcam, Cambridge, UK (OCN, ALP, OPN, Runx2, COL-1, ANG-1, VEGF, and HIF-1α).

### Rat critical-sized calvarial defect model

All procedures involving animals were approved by the Animal Research Committee of Peking Union Medical College Hospital (XHDW-2020-038). The Guide for the Care and Use of Laboratory Animals (GB14925-2010; NIH), and the Laboratory Animal Center of Peking Union Medical College Hospital were strictly adhered to. To maintain the overall health of the experimental animals, a series of measures were employed, including actively improving the feeding environment and regular use of prophylactic antibiotics. All rats were housed in a light- and temperature-controlled environment with free access to food and water. Sixteen male rats (eight weeks old) were randomly allocated into four groups: PBS (Control), BMSC-Exo, BMSC-Fe_3_O_4_-Exo and BMSC-Fe_3_O_4_-SMF-Exo. The rats were anesthetized by intraperitoneal administration of 50 mg/kg pentobarbital sodium (Sigma-Aldrich) before operation. Under sterile conditions, a 1.0- to 1.5-cm midline sagittal incision was made on the scalp, and the calvarium was exposed by blunt dissection. A critical-size defect (5 mm in diameter) was created in the middle of the parietal bone using a sterile drill. A pie-shaped piece (φ diameter = 5 mm and height = 2 mm) of porous absorbable surgical gelfoam (the average pore size = 500 μm) was added with 100 μL PBS or one type of exosomes (200 μg exosomes dissolved in 100 μL of PBS), and then implanted into the bone defect. At last, the incision was sutured with silk thread.

### Micro-CT analysis

The animals were euthanized 12 weeks postoperatively, and their skulls were explanted and fixed in 4% paraformaldehyde. The morphology of the reconstructed skulls was assessed using micro-CT to determine the bone volume. The percentage of new bone volume relative to tissue volume (BV/TV), the bone mineral density (BMD), and the trabecular number and thickness were determined using Mimics software (Materialise, Leuven, Belgium).

### Histological and immunofluorescence analysis

The collected rat crania were fixed in 10% paraformaldehyde solution, decalcified with 5% EDTA and embedded in paraffin. Sections from the mid-defect region were stained with hematoxylin and eosin (HE) and imaged under an optical microscope. Masson’s trichrome staining was used to evaluate the degree of collagen maturity.

For the immunohistochemical analysis, the sections were rehydrated, blocked, and incubated with primary anti-OCN or anti-α-SMA antibody (1:100; Abcam) at 4 °C overnight. After incubation with the secondary antibody (1:250; Abcam) at room temperature, the stained sections were visualized using the DAB substrate and finally counterstained with hematoxylin. Immunofluorescence staining for Runx2 or CD31 was performed to estimate the extent of newly formed bone and capillaries. Sections were incubated with anti-Runx2 or anti-CD31 antibodies (1:100; Abcam) overnight at 4 °C and then with the secondary antibody at room temperature for 1 h in the dark. All images were examined by another experienced histologist in a blinded manner.

### RNA sequencing and bioinformatics analysis

The miRNA expression profiles were compared between BMSC-Exos and BMSC-Fe_3_O_4_-SMF-Exos after they were determined by small RNA sequencing. Briefly, total RNA was extracted from exosomes, and cDNA and small sequencing libraries were prepared according to the Illumina sequencing protocol. The expression levels of miRNAs were estimated by comparing the sequencing data to a bioinformatics miRNA database and corrected by the number of reads per million. Candidate target genes of miRNAs were predicted by the online tools TargetScan, miRanda and miRWalk. Kyoto Encyclopedia of Genes and Genomes (KEGG) pathway enrichment analysis was performed for candidate target genes related to osteogenesis or angiogenesis. 

### Luciferase reporter assay

The wild-type (wt) and mutant (mut) 3′-UTRs of HDAC7 or COL4A2 were amplified by PCR and inserted into the pGL3 plasmid. HEK293 cells (5 × 10^4^ cells/well) were seeded in 48-well plates and co-transfected with the wt or mut luciferase reporter (100 ng) and miR-1260a mimics (20 nM) or with negative controls (NCs) as indicated. After 48 h, the relative luciferase activity was measured using a luciferase reporter system (Promega, Madison, WI, USA).

### Cell transfection

Cells were transfected using Lipofectamine 3000 (Invitrogen). MiR-1260a mimics or inhibitors and their NCs (RiboBio, Guangzhou, China) were transfected into BMSCs and HUVECs to evaluate miR-1260a function. After 48 h of transfection, the level of miR-1260a was measured by qRT-PCR. Overexpression of HDAC7 and COL4A2 was achieved by transfecting cells with HDAC7 or COL4A2 cDNA (Wei Zheng, Shandong, China) using Lipofectamine 3000.

### Statistical analysis

All experiments were performed with at least three replicates per group. The data shown are representative of these experiments and are presented as the mean ± standard deviation. Multiple group comparisons were performed by two-way analysis of variance with Tukey’s post hoc test. Statistical analysis was conducted using GraphPad Prism 7.0 software, and statistical significance was declared as (*) *p* < 0.05, (**) *p* < 0.01 and (***) *p* < 0.001.

## Results

### Magnetic conditions

To determine the best stimulation conditions (the concentration of Fe_3_O_4_ nanoparticles & the magnetic strength of SMF), BMSCs were separately cultured in the medium containing 0, 25, 50, 100, and 200 µg/mL Fe_3_O_4_ nanoparticles with SMFs of different strengths (0, 50, 100, and 200 mT). The results of CCK-8 assays revealed that the condition of 50 µg/mL Fe_3_O_4_ nanoparticles and a 100mT SMF was more suitable for the growth and proliferation of BMSCs (Fig. 1b, c). Therefore, 50 µg/mL Fe_3_O_4_ and a 100mT SMF were chosen as the optimum concentration and strength for the following studies. The TEM results showed that the MNPs were distributed in the cell nucleus and cytoplasm after they were taken up by BMSCs, and there was no significant change in the morphology of BMSCs (Fig. 1d).

### Characterization and internalization of exosomes

**Fig. 2:**
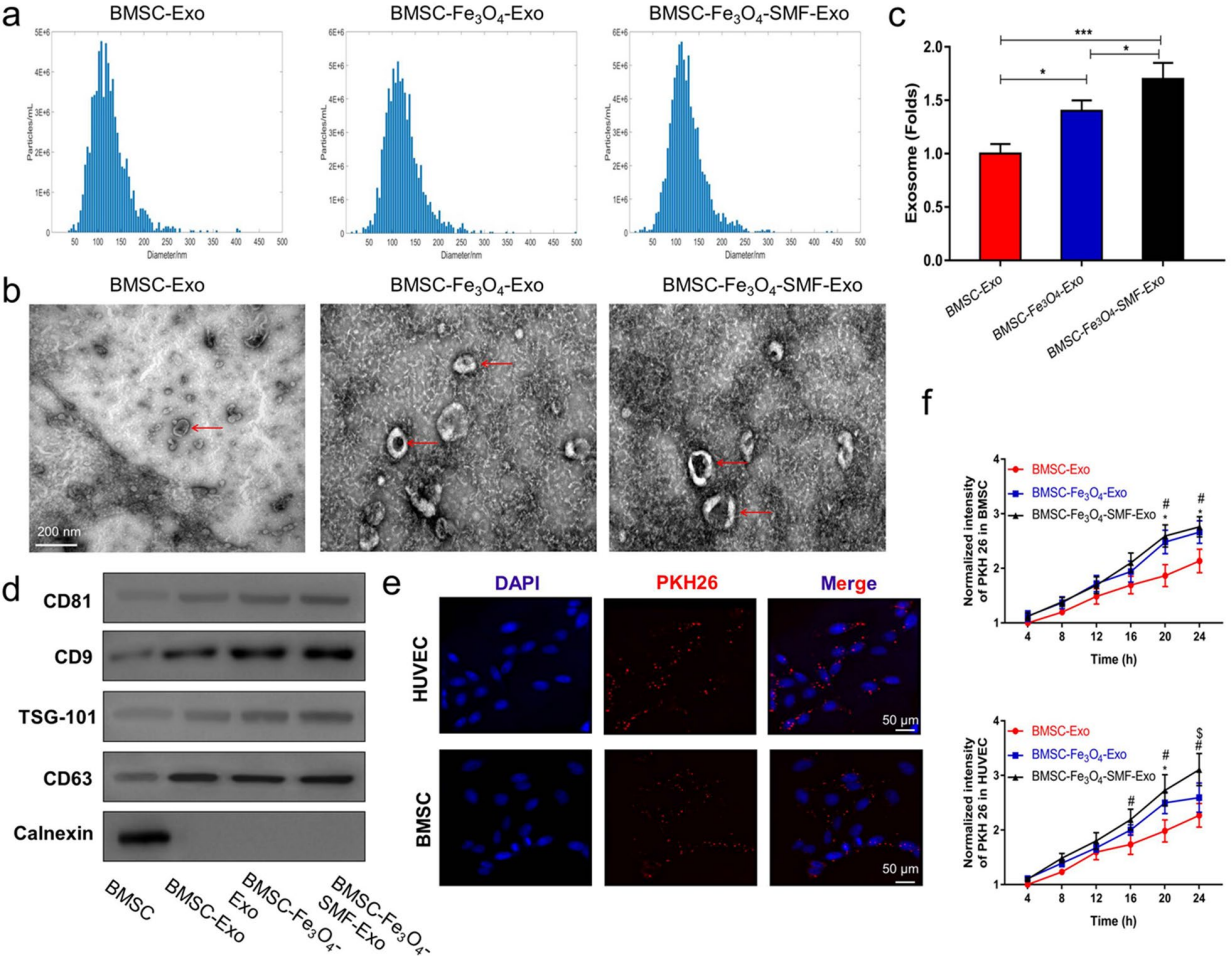
Characterization and internalization of three types of exosomes. **a** NTA analysis of three types of exosomes reveals that they exhibit similar size ranges. **b** Morphology of three types of exosomes under TEM; the red arrows indicate exosomes. **c** Fe_3_O_4_ nanoparticles and SMF increase the production of exosomes in BMSCs. (*) *p* < 0.05, (***) *p* < 0.001. **d** Western blotting analysis of the exosomal proteins CD9, CD63, CD81, TSG101 and the negative marker Calnexin. **e** Uptake of red fluorescent dye (PKH26)-labeled BMSC-Fe_3_O_4_-SMF-Exos by BMSCs and HUVECs. **f** Statistical evaluation of fluorescence intensities at different times. (*) significant differences between the BMSC-Exo group and BMSC-Fe_3_O_4_-Exo group (*p* < 0.05), (#) significant differences between the BMSC-Exo group and BMSC-Fe_3_O_4_-SMF-Exo group (*p* < 0.05), ($) significant differences between the BMSC-Fe_3_O_4_-Exo group and BMSC-Fe_3_O_4_-SMF-Exo group (*p* < 0.05)

Then, we characterized and quantified the three previous isolated exosomes. The results of the NTA analysis showed that the size of the particles in all three groups of exosomes predominantly ranged from 52 to 168 nm (Fig. 2a). All three exosomes have a similar cup- or sphere-shaped morphologies as TEM images shown in Fig. 2b. Interestingly, there was no significant differences in size or shape were observed among the three types of exosomes. Moreover, the yield of exosomes was significantly higher in the BMSC-Fe_3_O_4_-SMF-Exo and BMSC-Fe_3_O_4_-Exo groups than in the BMSC-Exo group (Fig. 2c), and the BMSC-Fe_3_O_4_-SMF-Exo group contained more exosomes than the BMSC- Fe_3_O_4_-Exos group (*p* < 0.05). The results of western blotting analysis further confirmed that all the exosomes had present exosome specific markers (CD9, CD63, CD81, and TSG101), whereas Calnexin (a negative marker) was absent (Fig. 2d).

To observe whether the three types of exosomes could be differentially taken up by BMSCs and HUVECs, exosomes were co-cultured with BMSCs or HUVECs for 24 h after they were labeled with PKH26. The rates of exosomes uptake by BMSCs or HUVECs were monitored using fluorescence microscopy in real time (Fig. 2e). The normalized intensities of PKH26 in BMSCs (20 h and 24 h) or HUVECs (16 h, 20 h and 24 h) were significantly higher in the BMSC-Fe_3_O_4_-SMF-Exo and BMSC-Fe_3_O_4_-Exo groups than those in the BMSC-Exo group (*p* < 0.05; Fig. 2f). These results suggested that BMSC-Fe_3_O_4_-SMF-Exos and BMSC-Fe_3_O_4_-Exos were taken up more easily than BMSC-Exos by BMSCs and HUVECs.

### BMSC-Fe_3_O_4_-SMF-Exos enhanced osteogenesis in vitro

**Fig. 3:**
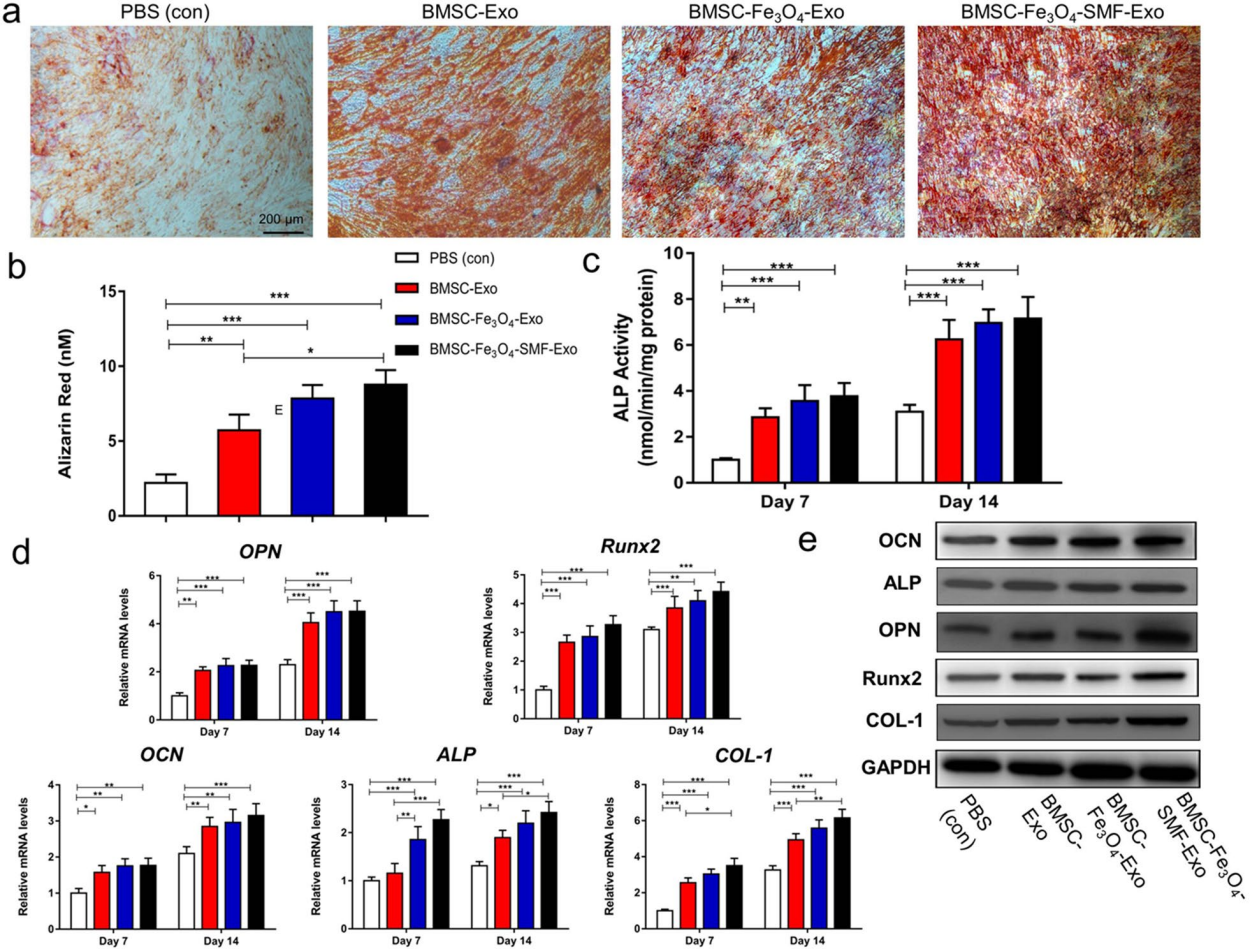
Enhanced osteogenic effect of exosomes by magnetic stimulation of BMSCs. (*) *p* < 0.05, (**) *p* < 0.01, (***) *p* < 0.001. **a**, **b** Osteogenic differentiation was assessed by ARS after 14 days incubation of BMSCs with exosomes and quantitative analysis. **c** Quantification of ALP staining after incubation of BMSCs with exosomes for 7 and 14 days. **d** mRNA expression levels of *OPN*, *Runx2*, *OCN*, *ALP* and *COL-1*. **e** Western blotting assay for the protein expression of OPN, Runx2, OCN, ALP and COL-1

The results of ARS showed that all the three exosomes could enhance mineral deposition of BMSCs compared with PBS (Fig. 3a). The quantitative result further revealed that there was significantly more calcium accumulation in the BMSC-Fe_3_O_4_-SMF-Exo group than in the BMSC-Exo group and the BMSC-Fe_3_O_4_-Exo group (*p* < 0.05; Fig. 3b). Then, ALP activity, ﻿a marker of early-stage osteogenic differentiation of BMSCs, was determined at days 7 and 14. The ALP levels in all three exosome groups were significantly higher than that in the control group at days 7 and 14 (*p* < 0.05; Fig. 3c). Two new exosomes could further improve the ALP activities of BMSCs compared with BMSC-Exos, and the effect of BMSC-Fe_3_O_4_-SMF-Exos was better than BMSC-Fe_3_O_4_-Exos. At last, qRT-PCR and western blotting were used to directly assess the gene expression levels and protein levels of the osteogenic markers (*OPN*, *Runx2*, *OCN*, *ALP,* and *COL-1*), respectively. The results showed that all the mRNA expression levels gradually increased with time from day 7 to 14, and all three types of exosomes significantly enhanced the expression of these osteogenic genes compared with PBS (*p* < 0.05; Fig. 3d). The largest increases of mRNA levels were observed in the BMSC-Fe_3_O_4_-SMF-Exo group. The protein levels of OPN, Runx2, OCN, ALP, and COL-1 were also markedly increased after treatment with BMSC-Fe_3_O_4_-SMF-Exos and BMSC-Fe_3_O_4_-Exos compared with BMSC-Exos at day 14 (Fig. 3e). All these results revealed that all the three exosomes derived from BMSCs could promote osteogenic differentiation of BMSCs, and BMSC-Fe_3_O_4_-SMF-Exos performed better than BMSC-Fe_3_O_4_-Exos and BMSC-Exos.

### BMSC-Fe_3_O_4_-SMF-Exos enhanced angiogenesis in vitro

**Fig. 4:**
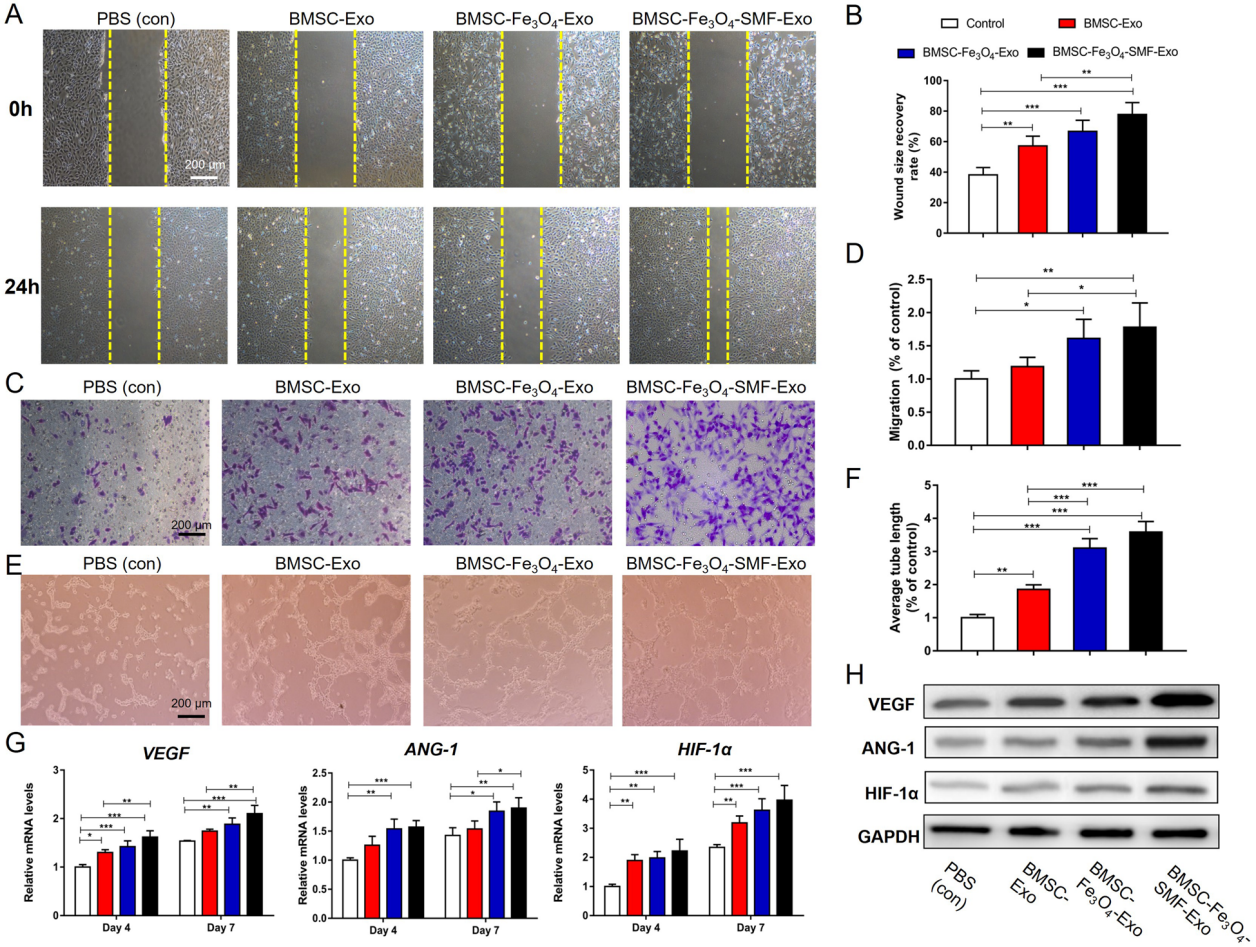
Magnetic stimulation enhances the angiogenic effect of exosomes in HUVECs. (*) *p* < 0.05, (**) *p* < 0.01, (***) *p* < 0.001. **a**, **b** Assessment of the migratory activity of HUVECs at 24 h by scratch wound assay and quantitative analysis of the wound recovery rate; the yellow dashed lines are the edges of the cell migration. **c**, **d** Transwell assay and quantitative analysis of the cell migration rate. **e**, **f** Tube formation by HUVECs and quantitative analysis of the average tube length. **g** mRNA expression levels of *VEGF*, *ANG-1* and *HIF-1α*. **h** Western blotting assay of the protein expression of VEGF, ANG-1 and HIF-1α

To determine the effects of the three types of exosomes on pro-angiogenic activity, scratch wound, transwell and tube formation assays were first performed. As shown in Fig. 4a–d, HUVECs co-cultured with exosomes migrated faster than those cocultured with PBS after incubation for 24 h, and HUVECs in the BMSC-Fe_3_O_4_-SMF-Exos group migrated noticeably faster than those in the other two exosome groups (*p* < 0.05). Furthermore, HUVECs co-cultured with BMSC-Fe_3_O_4_-SMF-Exos or BMSC-Fe_3_O_4_-Exos could generate more cord-like structures on Matrigel than those co-cultured with BMSC-Exos or PBS (Fig. 4e, f). Then, the expressions of angiogenic genes (*VEGF*, *ANG-1,* and *HIF-1α)* were measured at the mRNA by qRT-PCR and protein levels by western blotting. The mRNA expression levels of all three genes increased gradually with time from day 4 to day 7. Their mRNA expressions were substantially upregulated in the three exosome-treated groups compared to the PBS group, and they were highest in the BMSC-Fe_3_O_4_-SMF-Exo group (Fig. 4g). Similarly, the western blotting results indicated that the protein levels of VEGF, ANG-1, and HIF-1α increased markedly after treatment with exosomes, and BMSC-Fe_3_O_4_-SMF-Exos enhanced the expression of these proteins more significantly than either BMSC-Fe_3_O_4_-Exos or BMSC-Exos (Fig. 4h). Collectively, these findings revealed that exosomes derived from BMSCs could enhance angiogenesis in vitro and BMSC-Fe_3_O_4_-SMF-Exos had the most significant effect on angiogenesis in vitro.

### Exosomes promote bone regeneration and angiogenesis in vivo

**Fig. 5:**
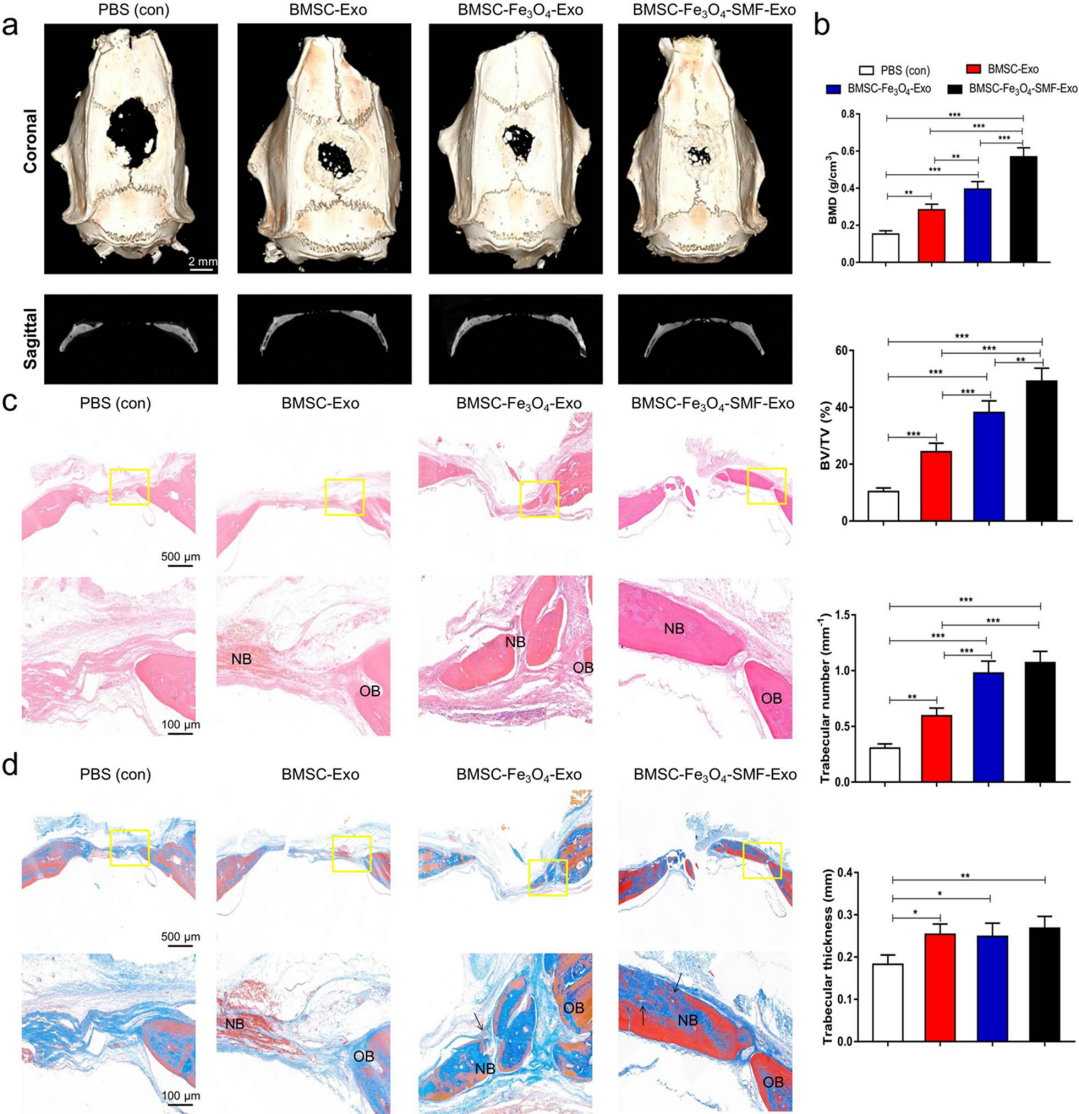
Exosomes from cells treated with magnetic stimulation increased bone formation in critical-sized rat calvarial defects. (*) *p* < 0.05, (**) *p* < 0.01, (***) *p* < 0.001. **a** Coronal (upper panel) and sagittal (lower panel) micro-CT images of bone formation in each group after 12 weeks. **b** Quantitative comparison of BMD, BV/TV ratio, trabecular number and trabecular thickness in the different groups. **c**, **d** HE staining and Masson’s trichrome staining in the four groups. The lower panels are the magnification of yellow box; the black arrows indicate the vascular structures and red marrow aggregation; OB, old bone; NB, new bone

Throughout the experiment, none of the experimental animals exhibited signs of anorexia, diarrhea, listlessness, or infection. In a critical-sized calvarial defect rat model, the morphology of newly formed bone was reconstructed by micro-CT. In the coronal and sagittal views, more newly formed bone filling the defect areas were observed in the BMSC-Fe_3_O_4_-SMF-Exo and BMSC-Fe_3_O_4_-Exo groups than in the BMSC-Exo and control groups, and the amount of newly formed bone was largest in the BMSC-Fe_3_O_4_-SMF-Exo group (Fig. 5a). According to quantitative analysis of the newly formed bone, BMD, BV/TV ratio and trabecular number were all markedly higher in the BMSC-Fe_3_O_4_-SMF-Exo and BMSC-Fe_3_O_4_-Exo groups than in the other two groups, indicating that the presence of exosomes released from BMSCs stimulated by Fe_3_O_4_ and SMF improved bone healing capacity in vivo. The trabecular thickness of the newly formed bone in defects displayed greater in all three exosome groups than in the PBS group (*p* < 0.05), but no significant differences among the three exosome groups (Fig. 5b).

The results of HE staining indicated that the bone defects in the control group were mainly filled with fibrotic connective tissue; in contrast, newly formed bone tissue was observed both along the border and in the center of the defects after administration of exosomes, and more newly formed bone could be observed in the BMSC-Fe_3_O_4_-SMF-Exo and BMSC-Fe_3_O_4_-Exo groups (Fig. 5c). The results of masson staining showed more collagen formation in the BMSC-Fe_3_O_4_-SMF-Exo than in the other three groups. In addition, more vasculature structures could be found around the newly formed bone in the BMSC-Fe_3_O_4_-SMF-Exo and BMSC-Fe_3_O_4_-Exo groups (Fig. 5d). These results showed the same trend as the results of micro-CT analysis and further supported the findings regarding bone formation.

**Fig. 6:**
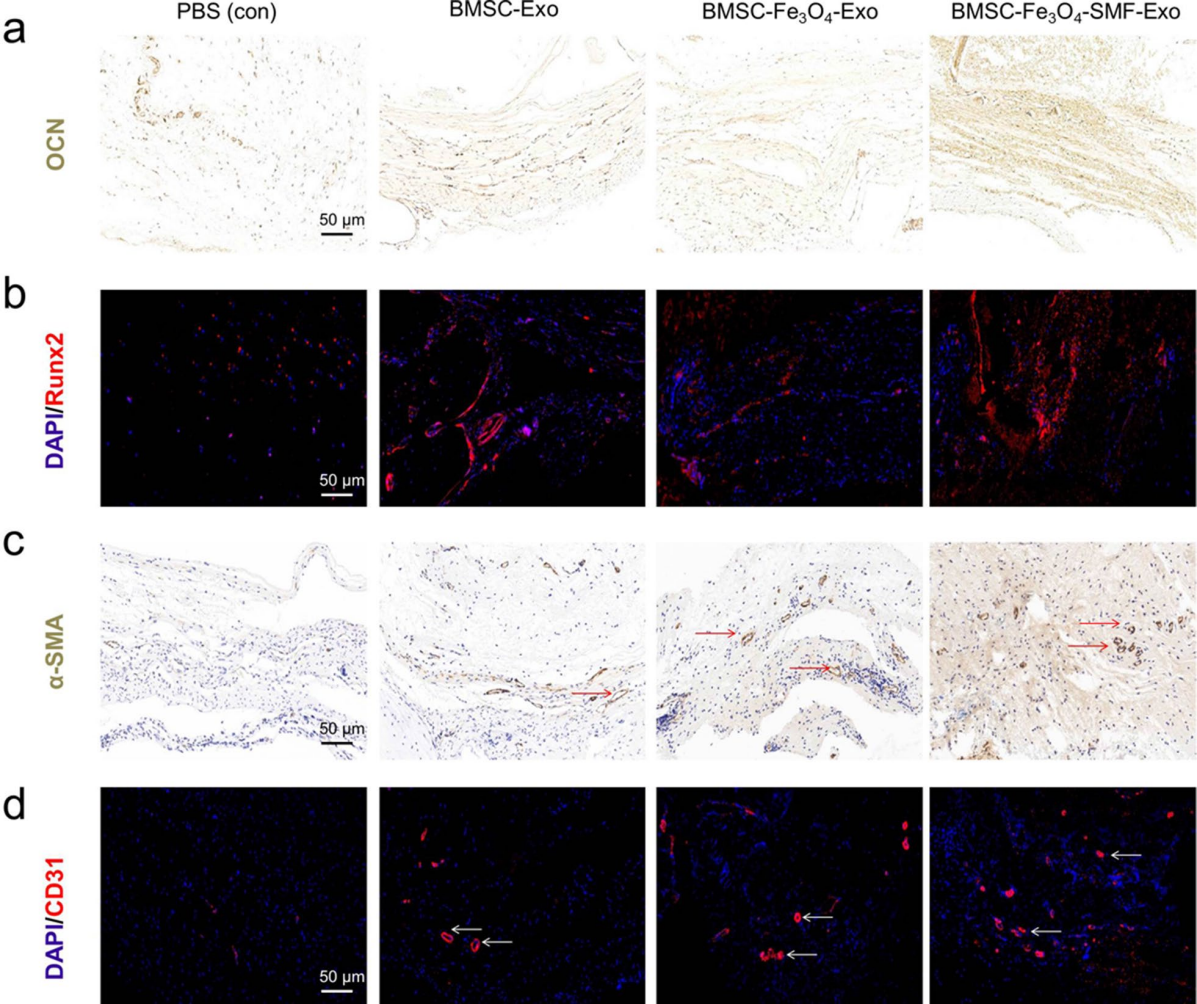
Immunohistochemical staining and immunofluorescence images showing that exosomes from cells treated with magnetic stimulation promote osteogenesis and angiogenesis. **a**, **b** Immunohistochemical staining of the osteogenic marker OCN and immunofluorescence analysis of the osteogenic marker Rnux2. **c**, **d** Immunohistochemical staining of the angiogenic marker α-SMA and immunofluorescence analysis of the angiogenic marker CD31. Dark brown granules indicating positive staining are marked by red arrows, and white arrows mark the newly formed vessels

The results of immunohistochemical staining for OCN showed that there were more OCN ( +) cells observed in the three exosome groups, and the most obvious OCN ( +) cells were found in the BMSC-Fe_3_O_4_-SMF-Exo group (Fig. 6a). The results of immunofluorescence staining for Rnux2 confirmed that bone regeneration in calvarial defects was enhanced by treating with exosomes, especially for BMSC-Fe_3_O_4_-SMF-Exos (Fig. 6a). Both results of immunohistochemical staining for α-SMA and immunofluorescence staining for CD31 revealed that some new vessels in the center area of bone defects could be observed in all three exosome groups, but rarely in the PBS group. There were more new vessels in the BMSC-Fe_3_O_4_-SMF-Exo group than in the other two exosome groups (Fig. 6c, d). These results further confirmed that exosomes can promote bone regeneration and angiogenesis in vivo, especially for BMSC-Fe_3_O_4_-SMF-Exos.

### miR-1260a is upregulated in BMSC-Fe_3_O_4_-SMF-Exos

**Fig. 7:**
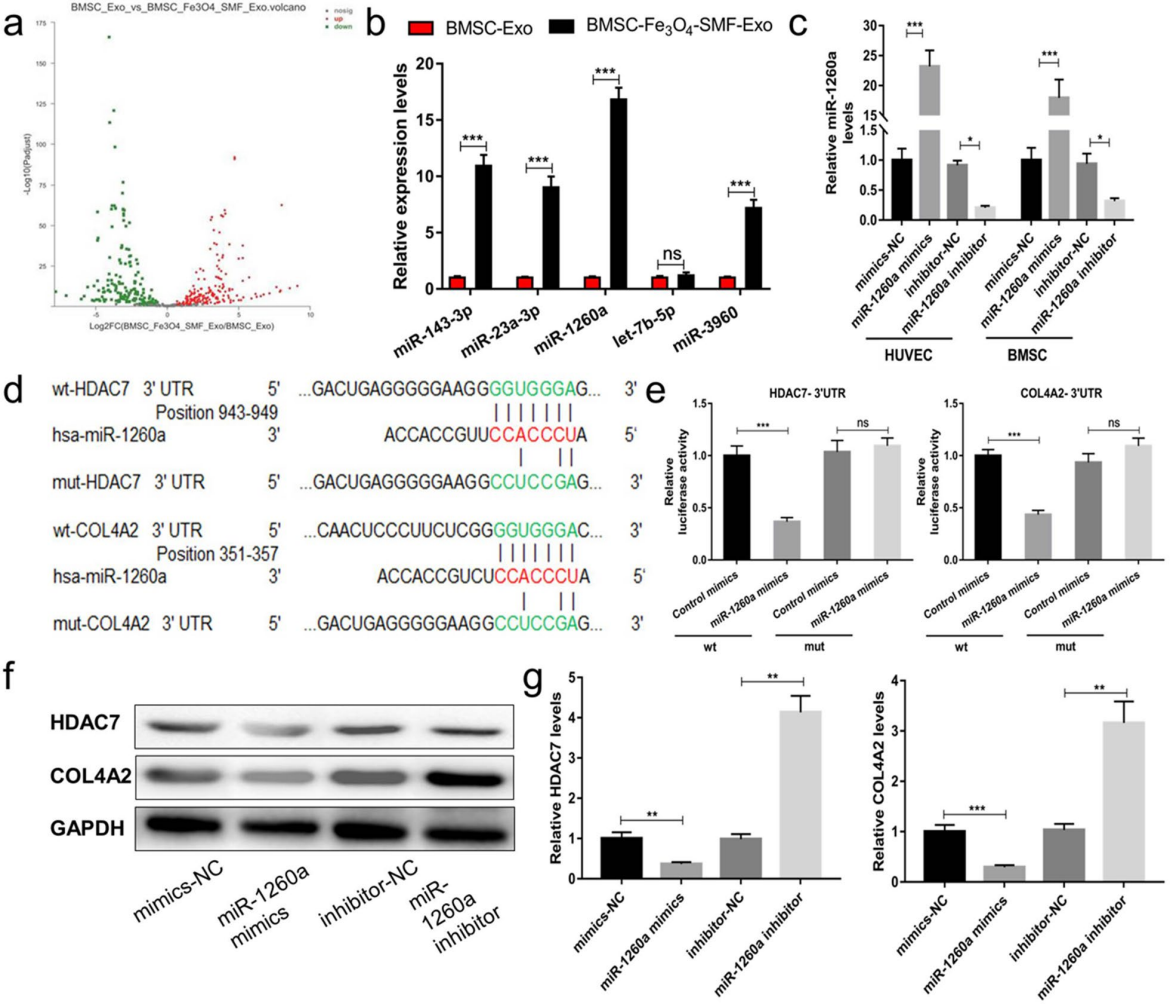
Exosomal miR-1260a derived from BMSC-Fe_3_O_4_-SMF-Exos regulates HDAC7 and COL4A2. (*) *p* < 0.05, (**) *p* < 0.01, (***) *p* < 0.001, ns = no significance; wt, wild-type; mut, mutant; NC, negative control. **a** Volcano plot of miRNA sequencing analysis of mRNAs with a ≥ 1.5-fold difference in expression between BMSC-Exos and BMSC-Fe_3_O_4_-SMF-Exos. Green and red indicate downregulation and upregulation, respectively. **b** Comparison of the top five elevated miRNAs (miR-143-3p, miR-23a-3p, miR-1260a, let-7b-5p and miR-3960) between BMSC-Exos and BMSC-Fe_3_O_4_-SMF-Exos using qRT-PCR. **c** Confirmation of the transfection efficiency of miR-1260a in HUVECs and BMSCs. **d** The miR-1260a binding sequence in the 3′-UTR of HDAC7 and COL4A2. **e** Luciferase readout from wt or mut HDAC7 3′-UTR co-transfected in BMSCs (left panel) or COL4A2 3′-UTR reporter co-transfected in HUVECs (right panel) with control mimics or miR-1260a mimics. The miR-1260a mimics transfection reduces luciferase activity when compared to control mimics transfection, which confirms that HDAC7 and COL4A2 are the target genes of miR-1260a. **f** The protein expression of HDAC7 and COL4A2 after transfection of cells with miR-1260a mimics, miR-1260a inhibitor and their NCs. **g** Transfection with the miR-1260a mimics resultes in a significant decrease in the levels of HDAC7 in BMSCs (left panel) and in the levels of COL4A2 in HUVECs (right panel)

To detect the potential molecular mechanism of BMSC-Fe_3_O_4_-SMF-Exos, miRNA sequencing analysis was first performed for BMSC-Exos and BMSC- Fe_3_O_4_-SMF-Exos. The volcano plot (Fig. 7a) showed that 181 miRNAs were upregulated and 148 miRNAs were downregulated in the BMSC-Fe_3_O_4_-SMF-Exo group compared to the BMSC-Exo group (≥ 1.5-fold, *p* < 0.05). Based on this miRNA profiling data, we selected the top five upregulated miRNAs (miR-143-3p, miR-23a-3p, miR-1260a, let-7b-5p and miR-3960) and further validated their expression using qRT-PCR. As shown in Fig. 7b, four of the five selected miRNAs (miR-143-3p, miR-23a-3p, miR-1260a, and miR-3960) were significantly upregulated in BMSC-Fe_3_O_4_-SMF-Exos compared to BMSC-Exos (*p* < 0.001). According to the results of miRNA sequencing and KEGG pathway enrichment analysis, as well as previous findings in the literatures [40–42], miR-1260a has positive effects on angiogenesis and osteoblast differentiation. Therefore, we chose miR-1260a as a potential key factor to check whether it was related to the promotion effect of BMSC-Fe_3_O_4_-SMF-Exos on osteogenesis and angiogenesis.

To verify that miR-1260a in BMSC-Fe_3_O_4_-SMF-Exos can be transferred to BMSCs and HUVECs via exosomes, we then measured miR-1260a levels in BMSCs and HUVECs treated with BMSC-Fe_3_O_4_-SMF-Exos or BMSC-Exos. An increase in the cellular level of mature miR-1260a but not pri-/pre-miR-1260a was observed in recipient BMSCs and HUVECs following treatment with BMSC-Fe_3_O_4_-SMF-Exos (Additional file 1: Fig. S1a, b). In addition, the increase of miR-1260a in BMSCs and HUVECs exposed to BMSC-Fe_3_O_4_-SMF-Exos was not prevented by an inhibitor of RNA polymerase II (Additional file 1: Fig. S1c). Cells were transfected with miR-1260a mimics or inhibitor and their NCs, and the transfection efficiency was confirmed by qRT-PCR (Fig. 7c). The results showed that BMSC-Fe_3_O_4_-SMF-Exos containing miR-1260a were internalized by BMSCs and HUVECs.

### Exosomal miR-1260a regulates HDAC7 and COL4A2 by targeting the 3′-UTR

We predicted possible miR-1260a targets that contribute to osteogenic and angiogenic functions by exploring online databases. To confirm the direct binding between miR-1260a and the 3′-UTR of its predicted target genes HDAC7 and COL4A2, we conducted luciferase reporter assays using luciferase reporter plasmids containing wild-type or mutated HDAC7 3′-UTR or COL4A2 3′-UTR with the miR-1260a binding site (Fig. 7d). Transfection of BMSCs and HUVECs with miR-1260a mimics reduced luciferase activity compared to transfection with control mimics (Fig. 7e). Consistent with the results of the reporter assay, transfection with the miR-1260a mimics resulted in a significant decrease in the levels of HDAC7 in BMSCs and in the levels of COL4A2 in HUVECs (*p* < 0.001), and treatment with the miR-1260a inhibitor yielded the opposite results (*p* < 0.01) (Fig. 7f, g).

### Exosomal miR-1260a promotes osteogenesis and angiogenesis by targeting HDAC7 and COL4A2

**Fig. 8:**
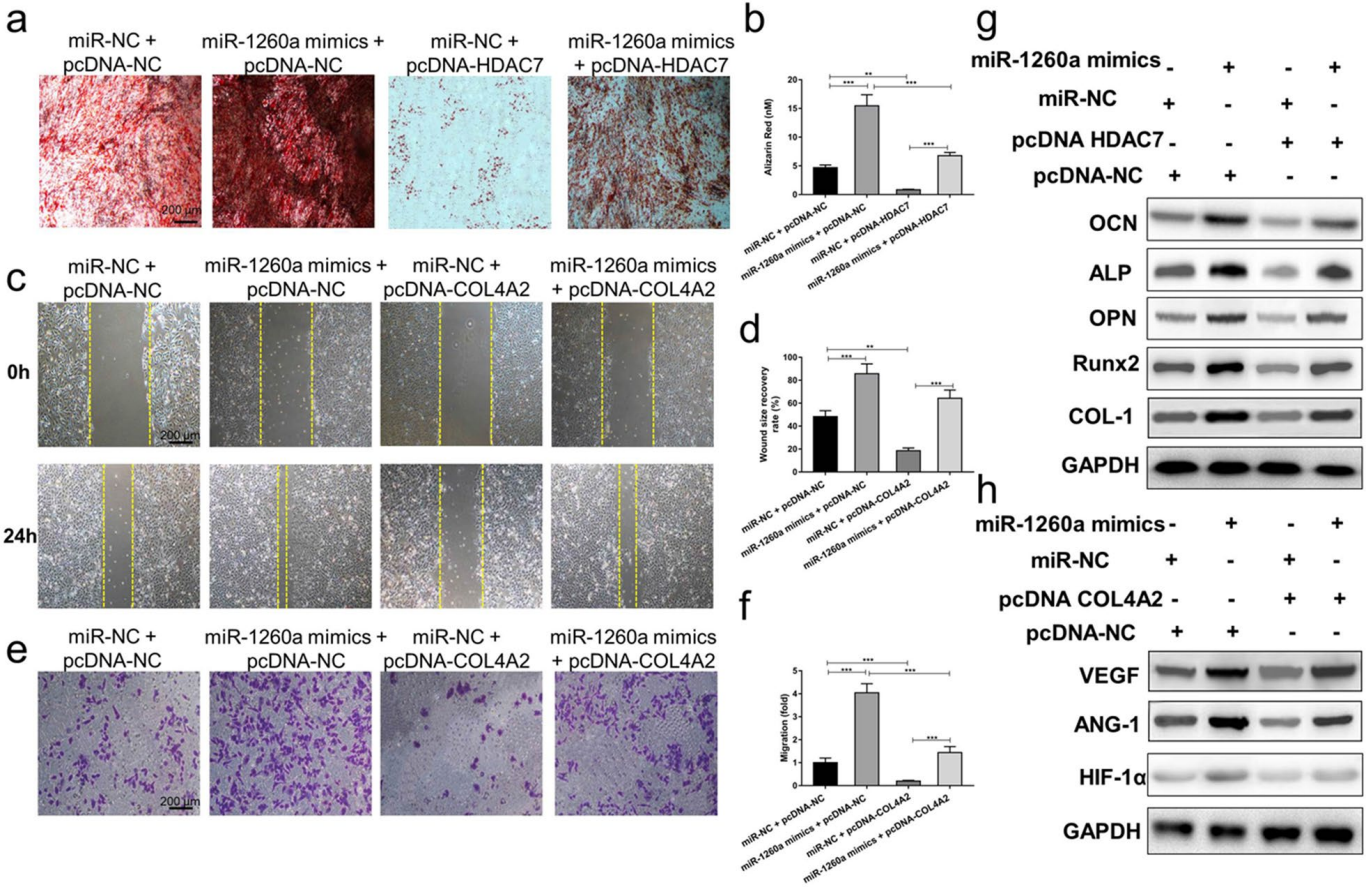
Exosomal miR-1260a promotes osteogenesis and angiogenesis by targeting HDAC7 and COL4A2. (**) *p* < 0.01, (***) *p* < 0.001, ns = no significance; NC, negative control. **a** The red staining indicative of mineralization is most obvious when BMSCs are cotransfected with miR-1260a mimics and pcDNA-NC, while matrix mineralization is significantly reduced when the BMSCs are cotransfected with miR-NC and pcDNA-HDAC7. **b** Quantitative analysis indicates overexpression of HDAC7 prevents the enhancement of osteoblast differentiation in BMSCs by miR-1260a mimics. **c** The scratch wound assay showing the areas of wound healing among different groups. **d** Quantitative analysis indicates overexpression of COL4A2 prevents the upregulation of the wound recovery rate of HUVECs by miR-1260a mimics. **e** The transwell assay showing the cell migration among different groups. **f** Quantitative analysis indicates overexpression of COL4A2 suppresses the upregulation of the migration rate of HUVECs by miR-1260a mimics. **g** Western blotting assays showing that overexpression of HDAC7 prevents the upregulation of OPN, Runx2, OCN, ALP and COL-1 protein expression by miR-1260a mimics. **h** Western blotting assays showing that overexpression of COL4A2 prevents the upregulation of VEGF, ANG-1 and HIF-1α protein expression by miR-1260a mimics

To further explore the relationship between exosomal miR-1260a and HDAC7 or COL4A2, a series of in vitro rescue experiments were conducted. We transfected miR-1260a mimics or miR-NC into BMSCs and HUVECs, then cotransfected the BMSCs with a plasmid that overexpresses HDAC7 (pcDNA-HDAC7) or with pcDNA-NC and cotransfected the HUVECs with a plasmid that overexpresses COL4A2 (pcDNA-COL4A2) or with pcDNA-NC. As shown in Fig. 8a, b, the red staining indicative of mineralization was most obvious when BMSCs were cotransfected with miR-1260a mimics and pcDNA-NC, while matrix mineralization was significantly reduced when the BMSCs were cotransfected with miR-NC and pcDNA-HDAC7. The results of this series of rescue experiments demonstrate that pcDNA-HDAC7 in BMSCs abolishes the promoting effect of exosomal miR-1260a mimics on osteoblast differentiation.

After 24 h cotransfection with miR-1260a mimics and pcDNA-NC, scratches made in the cell monolayer were completely covered by HUVECs, and the migration rate of the cells increased significantly, whereas after cotransfection with miR-NC and pcDNA-COL4A2, the areas of wound healing and the migration rate were the lowest (Fig. 8c–f). Collectively, the effect of miR-1260a mimics on enhancing angiogenesis in HUVECs was prevented by transfection of the cells with pcDNA-COL4A2.

**Fig. 9:**
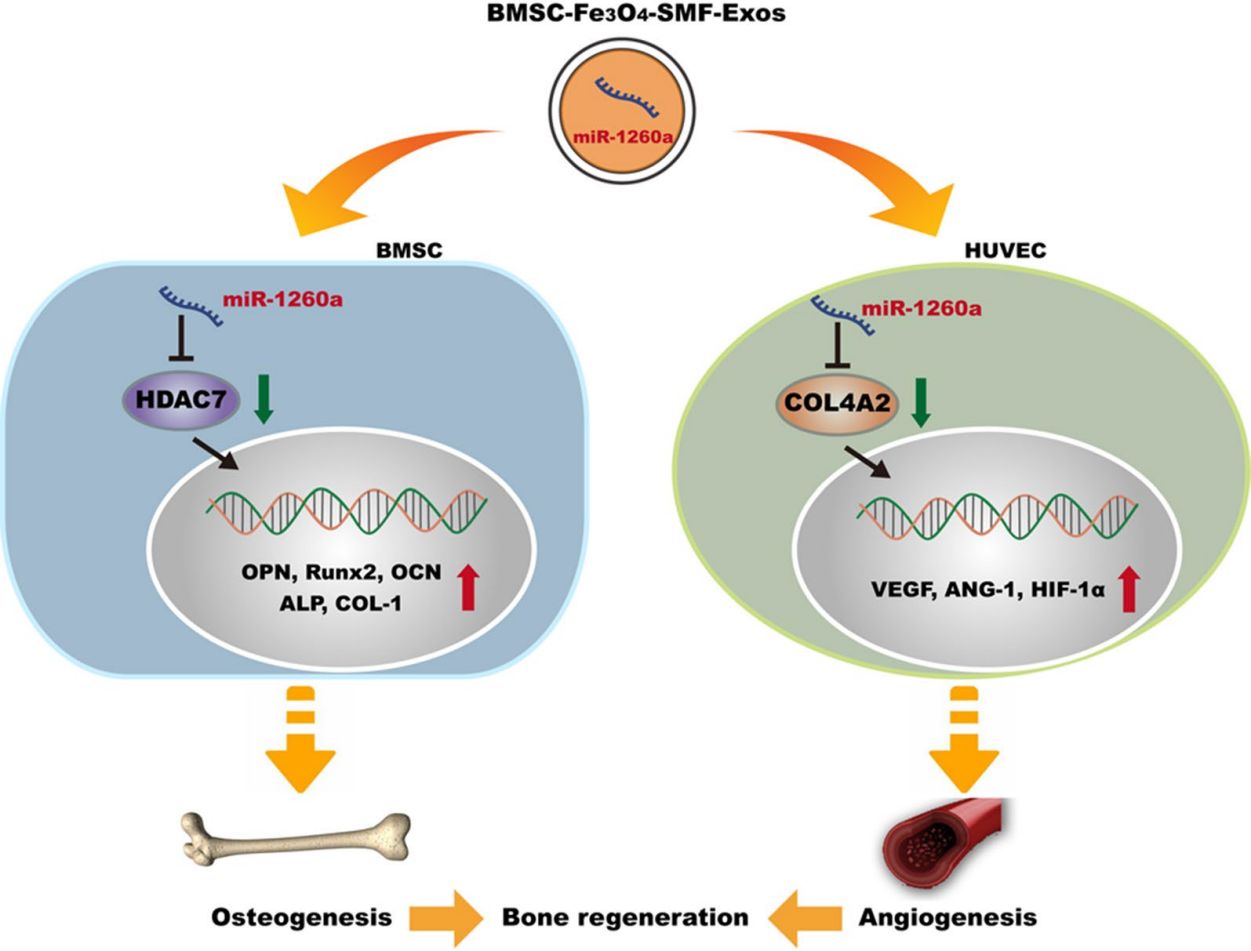
Schematic diagram depicting the detailed mechanisms involved in the improvement of osteogenesis and angiogenesis by miR-1260a derived from BMSC-Fe_3_O_4_-SMF-Exos

The western blotting assay also revealed that miR-1260a mimics enhanced the levels of osteogenic and angiogenic protein expression, whereas pcDNA-HDAC7 and pcDNA-COL4A2 attenuated these (Fig. 8g, h). The results of this series of rescue experiments demonstrated that pcDNA-HDAC7 in BMSCs and pcDNA-COL4A2 in HUVECs could abolish the promoting effect of exosomal miR-1260a mimics on osteogenesis and angiogenesis. Overexpression of HDAC7 rescued osteogenic activity, overexpression of COL4A2 rescued angiogenic activity, and both were enhanced by miR-1260a mimics. We concluded that exosomal miR-1260a derived from BMSC-Fe_3_O_4_-SMF-Exos promoted osteogenesis by targeting HDAC7 and that it promoted angiogenesis by targeting COL4A2 (Fig. 9).

## Discussion

Natural bone regeneration process requires orchestrated coupling between osteogenesis and angiogenesis, thus tissue engineering strategies to construct vascularized synthesized scaffold potentially revolutionize the treatment of critical size bone defects. Farshadi et al. found that SiC together with the nano-hydroxyapatite/gelatin scaffold where seeded with mesenchymal stem cells could be useful for bone repair [43]. Kazemi et al. demonstrated the gelatin/bioactive glass nanocomposite scaffold with endothelial cells could enhance bone regeneration and vascularization[44]. The dual-functional regulation for angiogenesis and osteogenesis has been recognized and many studies have focused on enhancing bone regeneration by administrating BMSCs [45, 46]. Recently BMSCs combined with biomaterials, performed a positive effect on tissue engineering. Li et al. utilized Ca^2+^-supplying black phosphorus-based scaffolds, nanofibers, and HA-porous SiO_2_ nanoparticles through microfluidic technology to cargo BMSCs for bone regeneration and tissue engineering [47]. Yang’s review described cell membrane-camouflaged biomimetic nanoparticles exhibited great potential in numerous biomedical applications [48]. Li et al. fabricated a novel 3D fibrous core–shell magnetic scaffold, which remarkably improved cellular proliferation and growth space [49]. Wang et al. reported the differentiation of BMSCs on the biocompatible, biodegradable, and biomimetic scaffolds promoted the regeneration of largely defected esophagus [50]. Jin et al. indicated that 3D chondroitin sulfate surface-modified silk nanofibers enhanced BMSCs adhesion and osteogenic differentiation [51]. Liao et al. concluded DUSP6 might increase the cell vitality of neural stem cells after Aβ treatment, probably via ERK1/2 activation [52]. Despite the therapeutic efficacy of BMSCs in bone regeneration, several problems still need to be solved and optimized to maintain the cell potency and viability. Exosomes (30–150 nm), formed by a continuous process of endocytosis, fusion, and excretion, play an important role in cell-to-cell communication during tissue repair. Compared with the direct use of stem cells, the utilization of exosomes overcomes many challenges and limitation, such as the time-consuming and dosage required, low survival rate of local transplanted cells, tumor formation and unwanted immune rejection [12].

The application of nano-structures for bone treatment has attracted extensive attention [53]. For example, using hydroxyapatite and biopolymer particles for fabrication of bone scaffolds displayed excellent bioactivity [54]. Our results revealed that exosomes derived from BMSCs after Fe_3_O_4_ nanoparticles and SMF stimulation robustly enhanced the proliferation, migration and tube formation of HUVECs, as well as the expression of pro-angiogenic factors, compared to those from untreated BMSCs. In addition, the results showed that, similar to BMSC-Fe_3_O_4_-SMF-Exos, BMSC-Fe_3_O_4_-Exos exhibited an elevated pro-angiogenic capacity. The pro-angiogenic potential is mainly attributed to the magnetic effects via stimulating exosomal miR-1260a secretion.

SMFs have been categorized according to their intensity as ultra-weak (5 µT to 1 mT), weak (1 mT), moderate (1 mT to 1 T), strong (1–5 T), and ultra-strong (> 5 T) [55]. Given the enhancing effects of magnetic fields on fracture healing, osteoarthritis and wound healing, the use of a SMF with moderate strength provided a noninvasive, safe, and easy method of treating the injured site [23, 56]; In addition, Fe_3_O_4_ nanoparticles are also typical magnetic materials for bone tissue regeneration. For example, Shuai et al. reported the construction of magnetic micro-environment in poly-_L_-lactide/polyglycolic acid (PLLA/PGA) scaffolds incorporating Fe_3_O_4_ MNPs could promote new bone tissue formation in vivo significantly [57]. And they concluded MNPs was conducive to enhancing cell activity on scaffolds and further promoting the growth of cells into the scaffolds, thus speeding up bone formation. To make the best use of these advantages, BMSCs stimulated by Fe_3_O_4_ nanoparticles and a SMF secreted the exosomes were used in the present study. Based on the concentration gradient experiments, 50 µg/mL Fe_3_O_4_ nanoparticles was selected as the optimal dose because addition of higher concentrations (> 100 µg/mL) resulted in decreased cell proliferation. Also, in our study, 100 mT SMF modulated cell proliferation and contributed to a marked increase in cell viability, especially when combined with a low dose of Fe_3_O_4_.

Both in vitro and in vivo analysis revealed that BMSC-Fe_3_O_4_-SMF-Exos enhanced osteogenesis and angiogenesis more effectively than BMSC-Exos. It is well recognized that miRNAs are one of the main functional components of exosomes and that they may play a crucial role in cell communication, eventually regulating biological functions. MiRNAs are a class of noncoding RNAs 18–24 nucleotides in length that post-transcriptionally downregulate gene expression; miRNAs downregulate gene expression by binding to the 3′-UTR of protein coding transcripts, resulting in either mRNA cleavage or translational repression [58–60]. In this study, we revealed that the abundance of miR-1260a is greatly increased in exosomes released by BMSCs preconditioned with Fe_3_O_4_ nanoparticles in combination with SMF and showed that the BMSC-Fe_3_O_4_-SMF-Exos could be taken up by BMSCs and HUVECs. In BMSCs, miR-1260a bound to the 3′-UTR of HDAC7 mRNA and directly inhibited its expression. Similarly, in HUVECs, the 3′-UTR of COL4A2 mRNA bound to miR-1260a, and the expression of COL4A2 was repressed by miR-1260a.

HDACs are conserved enzymes that remove acetyl groups from lysine side chains in histones. As described in the literature [61], HDAC7 represses Runx2 activity, and HDAC inhibitors accelerate osteoblast differentiation in vitro. For example, Wang et al. found that miR-143 promotes angiogenesis coupled with osteoblast differentiation by targeting HDAC7 [62]. COL4 is the main component of the basement membrane extracellular matrix. Canstatin, a non-collagenous C-terminal fragment of the COL4A2 chain, was initially identified as an endogenous antiangiogenic factor [63, 64]. Collectively, the BMSC-Fe_3_O_4_-SMF-Exos tested in the current study promoted osteogenesis in a miR-1260a/HDAC7-dependent manner and enhanced angiogenesis through miR-1260a/COL4A2.

This study highlights the therapeutic potential of BMSC-Fe_3_O_4_-SMF-Exos and BMSC-Fe_3_O_4_-Exos, which may not only promote the synergic regulation for angiogenesis and osteogenesis, and represent an effective and promising protocol for the optimization of therapeutic actions for bone regeneration, but also act as biological vectors for the delivery of biologically functional miR-1260a into recipient cells. However, there were several limitations to the present study. First, it remains to be determined how Fe_3_O_4_ nanoparticles and SMF could induce BMSCs to release exosomes containing more miR-1260a than BMSCs with no interventions in further studies, that is the upstream genes functioned on the exosomal miR-1260a. Moreover, how to minimize the residual Fe_3_O_4_ in the BMSC-Fe_3_O_4_-Exos and BMSC-Fe_3_O_4_-SMF-Exos after isolation by ultracentrifugation was not further optimized in the present study.

## Conclusions

The present study demonstrated a novel phenomenon that, compared with BMSC-Exos, BMSC-Fe_3_O_4_-Exos and BMSC-Fe_3_O_4_-SMF-Exos promoted greater bone regeneration by improving osteogenesis and angiogenesis in vitro and in vivo. BMSC-Fe_3_O_4_-SMF-Exos exerted the most marked effect. It was confirmed that miR-1260a was upregulated in BMSC-Fe_3_O_4_-SMF-Exos and that exosomal miR-1260a enhanced osteogenesis and angiogenesis by suppressing HDAC7 and COL4A2 expression. Thus, low doses of Fe_3_O_4_ nanoparticles combined with SMF may trigger exosomes in a way that promotes their therapeutic effect and accelerates the bone defect healing process. Our findings provide novel insight into a process that may have therapeutic potential for bone regeneration in the future.

## Supplementary Information

**Additional file 1. **Additional tables and figures.

## Data Availability

Most of the datasets supporting the conclusions of this article are included within this article and the additional files. The datasets used or analyzed during the current study are available on reasonable request.
