# Correction: Bone mesenchymal stem cells stimulation by magnetic nanoparticles and a static magnetic field: release of exosomal miR-1260a improves osteogenesis and angiogenesis

**DOI:** 10.1186/s12951-023-01978-0

**Published:** 2023-07-10

**Authors:** Di Wu, Xiao Chang, Jingjing Tian, Lin Kang, Yuanhao Wu, Jieying Liu, Xiangdong Wu, Yue Huang, Bo Gao, Hai Wang, Guixing Qiu, Zhihong Wu

**Affiliations:** 1 Department of Orthopaedic Surgery, Peking Union Medical College Hospital, Peking Union Medical College and Chinese Academy of Medical Sciences, No. 1 Shuaifuyuan, Beijing, 100730 China; 2 Medical Science Research Center (MRC), Peking Union Medical College Hospital, Peking Union Medical College and Chinese Academy of Medical Sciences, No. 1 Shuaifuyuan, Beijing, 100730 China; 3 Umibio (Shanghai) Co. Ltd; RM309, 1st Building, No. 88 Cailun Rd, Shanghai, 201210 China; 4 Beijing Key Laboratory for Genetic Research of Bone and Joint Disease, No. 1 Shuaifuyuan, Beijing, 100730 China; 5 State Key Laboratory of Complex Severe and Rare Diseases, Peking Union Medical College Hospital, No. 1 Shuaifuyuan, Beijing, 100730 China

**Correction: J Nanobiotechnol (2021) 19:209** 10.1186/s12951-021-00958-6

Following publication of the original article [1], the authors identified some errors in Fig. 4a and c. The correct Fig. 4 is given in this erratum.

**Fig. 4:**
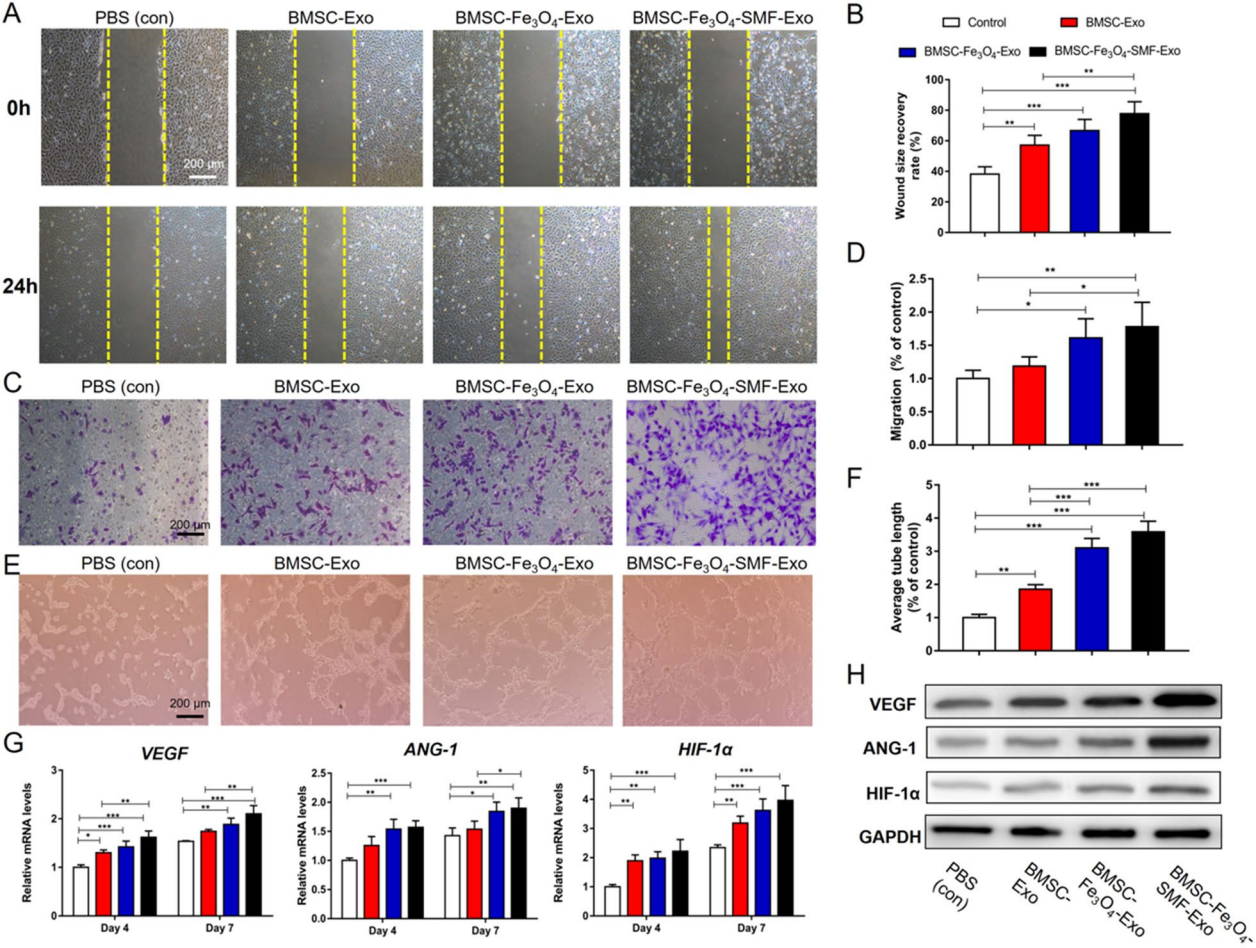
Magnetic stimulation enhances the angiogenic effect of exosomes in HUVECs. (*) p < 0.05, (**) p < 0.01, (***) p < 0.001. **a**, **b** Assessment of the migratory activity of HUVECs at 24 h by scratch wound assay and quantitative analysis of the wound recovery rate; the yellow dashed lines are the edges of the cell migration. **c**, **d** Transwell assay and quantitative analysis of the cell migration rate. **e**, **f** Tube formation by HUVECs and quantitative analysis of the average tube length. **g** mRNA expression levels of VEGF, ANG-1 and HIF-1α. **h** Western blotting assay of the protein expression of VEGF, ANG-1 and HIF-1α

This error does not affect the conclusions of this research. The authors apologize for not noticing this error before publication, and for any inconvenience caused. The original article has been corrected.
