# Supplementary material for: Bone mesenchymal stem cells stimulation by magnetic nanoparticles and a static magnetic field: release of exosomal miR-1260a improves osteogenesis and angiogenesis
Source: J Nanobiotechnology. 2021 Jul 13;19:209. doi: 10.1186/s12951-021-00958-6 (PMC8278669; doi:10.1186/s12951-021-00958-6)
Supplement: Supplementary file 1 — Additional file 1. Additional tables and figures. [file 12951_2021_958_MOESM1_ESM.docx]

Additional file 1: Table S1

| Gene | Primer Sequence, 5’–3’ | |
| --- | --- | --- |
|  | Forward | Reverse |
| *OPN*  *Runx2*  *OCN*  *ALP*  *COL-1*  *GAPDH*  *VEGF*  *ANG-1*  *HIF-1α*  *HDAC7*  *COL4A2*  *U6* | GAAGTTTCGCAGACCTGACAT  TGGTTACTGTCATGGCGGGTA  GGCGCTACCTGTATCAATGG  ACTGGGGCCTGAGATACCC  GAGGGCCAAGACGAAGACATC  ACAACTTTGGTATCGTGGAAGG  AGGGCAGAATCATCACGAAGT  AGCGCCGAAGTCCAGAAAAC  GAACGTCGAAAAGAAAAGTCTCG  TGCCCAGTCCTTAATGACCAC  TTATGCACTGCCTAAAGAGGAGC  TGGAACGCTTCACGAATTTGCG | GTATGCACCATTCAACTCCTCG  TCTCAGATCGTTGAACCTTGCTA  GTGGTCAGCCAACTCGTCA  TCGTGTTGCACTGGTTAAAGC  CAGATCACGTCATCGCACAAC  GCCATCACGCCACAGTTTC  AGGGTCTCGATTGGATGGCA  TACTCTCACGACAGTTGCCAT  CCTTATCAAGATGCGAACTCACA  CACCTGGACGTGAGTTTTGAG  CCCTTAACTCCGTAGAAACCAAG  GGAACGATACAGAGAAGATTAGC |

Additional file 1: Table S2

| miRNA | Primer sequence, 5’–3’ |
| --- | --- |
| hsa-miR-143-3p  hsa-miR-23a-3p  hsa-miR-1260a  hsa-let-7b-5p  hsa-miR-3960 | TGCAGTGCTGCATCTCT  AGATCACATTGCCAGGGAT  AGATCCCACCTCTGCC  CAGTGAGGTAGTAGGTTGTGT  CGGCGGCGGAG |


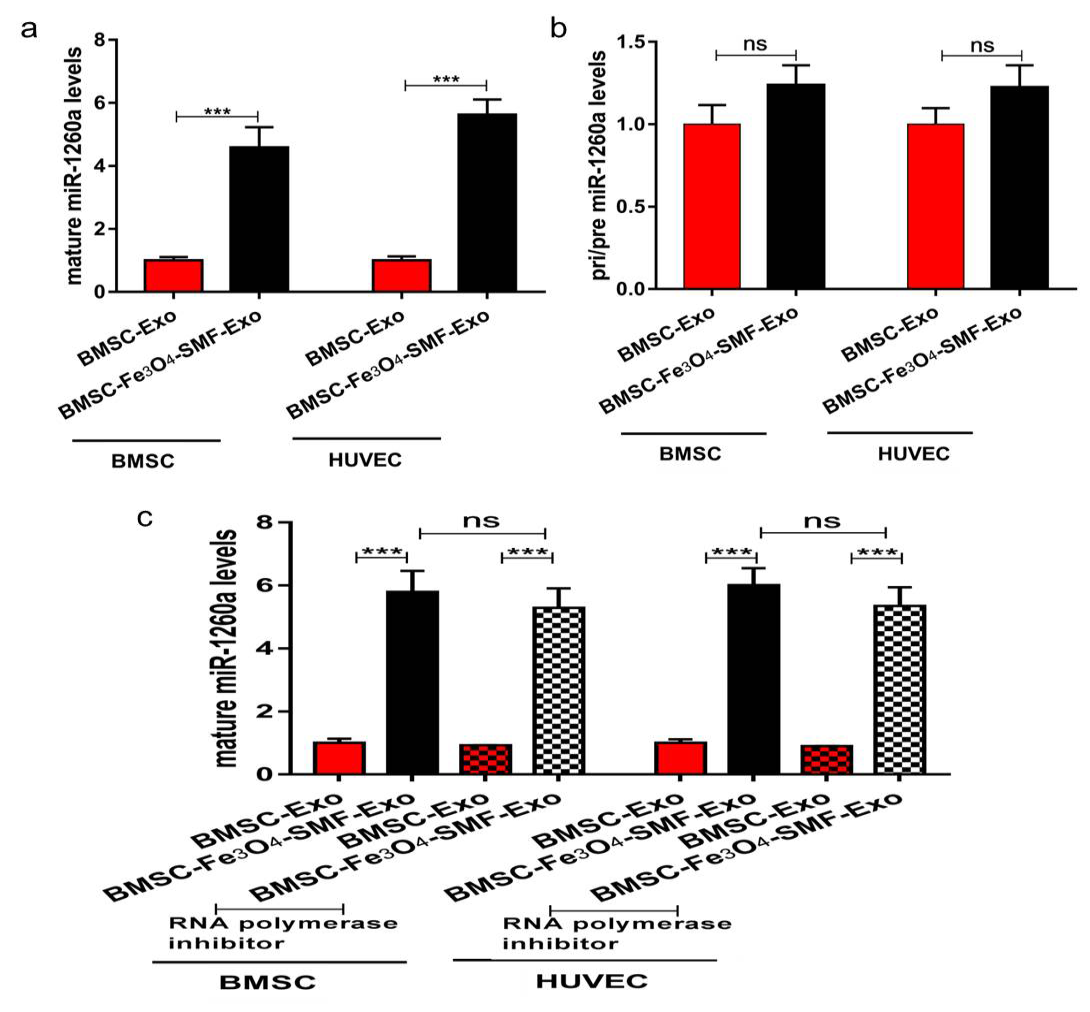


Figure S1: BMSC-Fe_3_O_4_-SMF-Exo-secreted miR-1260a can be transferred to BMSCs and HUVECs via exosomes. (***) *p* < 0.001, ns = no significance. (a, b) Levels of mature and pri/pre miR-1260a in exosome-treated BMSCs and HUVECs. (c) Treatment with an RNA polymerase II inhibitor did not alter the level of miR-1260a in BMSCs or HUVECs exposed to BMSC-Fe_3_O_4_-SMF-Exos.
